# Biodegradable magnesium-based screw clinically equivalent to titanium screw in hallux valgus surgery: short term results of the first prospective, randomized, controlled clinical pilot study

**DOI:** 10.1186/1475-925X-12-62

**Published:** 2013-07-03

**Authors:** Henning Windhagen, Kerstin Radtke, Andreas Weizbauer, Julia Diekmann, Yvonne Noll, Ulrike Kreimeyer, Robert Schavan, Christina Stukenborg-Colsman, Hazibullah Waizy

**Affiliations:** 1 Department of Orthopaedic Surgery, Hannover Medical School, Anna-von-Borries-Str.1-7, 30625 Hannover, Germany; 2 Syntellix AG, Schiffgraben 11, 30159 Hannover, Germany

**Keywords:** Magnesium, Degradable, Hallux valgus, Osteosynthesis

## Abstract

**Purpose:**

Nondegradable steel-and titanium-based implants are commonly used in orthopedic surgery. Although they provide maximal stability, they are also associated with interference on imaging modalities, may induce stress shielding, and additional explantation procedures may be necessary. Alternatively, degradable polymer implants are mechanically weaker and induce foreign body reactions. Degradable magnesium-based stents are currently being investigated in clinical trials for use in cardiovascular medicine. The magnesium alloy MgYREZr demonstrates good biocompatibility and osteoconductive properties. The aim of this prospective, randomized, clinical pilot trial was to determine if magnesium-based MgYREZr screws are equivalent to standard titanium screws for fixation during chevron osteotomy in patients with a mild hallux valgus.

**Methods:**

Patients (n=26) were randomly assigned to undergo osteosynthesis using either titanium or degradable magnesium-based implants of the same design. The 6 month follow-up period included clinical, laboratory, and radiographic assessments.

**Results:**

No significant differences were found in terms of the American Orthopaedic Foot and Ankle Society (AOFAS) score for hallux, visual analog scale for pain assessment, or range of motion (ROM) of the first metatarsophalangeal joint (MTPJ). No foreign body reactions, osteolysis, or systemic inflammatory reactions were detected. The groups were not significantly different in terms of radiographic or laboratory results.

**Conclusion:**

The radiographic and clinical results of this prospective controlled study demonstrate that degradable magnesium-based screws are equivalent to titanium screws for the treatment of mild hallux valgus deformities.

## Introduction

Currently, nondegradable implants are primarily made of steel or titanium. Although these implants provide maximum stability, these nondegradable materials interfere with imaging modalities, such as X-ray and magnetic resonance imaging, and often require an undesirable second operation to remove the implant [1,2]. Moreover, the mechanical properties of nondegradable implants (steel or titanium) are quite different from those of cortical bone, potentially resulting in inhomogeneous stress transfer and limiting the bone-healing process [3,4]. This constellation of effects defines “stress shielding.” Therefore, it might be beneficial to use implants material with a Young’s modulus close to that of cortical bone.

Currently, the most commonly used degradable implants are polymer-based. These are mechanically weaker than metallic devices and are associated with foreign body reactions and osteolysis [5]. However, the first magnesium-based implants used at the beginning of the 20th century exhibited high corrosion rates that consequently generated subcutaneous gas cavities and reduced mechanical stability [6]. Recently developed magnesium-based implants demonstrate improved anticorrosive and mechanical properties [1]. Degradable magnesium-based intravascular stents (WE43) yield good clinical results and are biocompatible [7].

The present study investigated the use of the MAGNEZIX® compression screw (Syntellix AG, Hannover, Germany). MAGNEZIX® is an aluminum-free magnesium alloy that is classified as an MgYREZr alloy according to DIN EN 1753. This alloy contains rare earth elements and is compositionally similar to WE43. It has already demonstrated good biocompatibility and osteoconductive quality *in vivo*[8].

The chevron osteotomy is a distal “V-shaped” metatarsal osteotomy that was first described by Austin and Leventen [9]. It is the operative option used to treat mild to moderate hallux valgus deformities. The angle of the “V” is about 60° and results in the impaction of the fragments without osteosynthesis. Recent studies have reported modification of the angulation and range of the limbs. The use of a greater angle and horizontal osteotomy can maximize the contact surface, but osteosynthesis would be necessary because fewer fragments would be impacted [10]. Loss of fixation and, consequently, malunions and pseudarthrosis, have been reported following surgery without fixation. Various studies have reported using K-wires, screws, staples, and plates [11]; however, screw fixation is mechanically superior to other modes of fixation [12].

The aim of this prospective, randomized clinical trial was to determine if the MgYREZr alloy-based screw (MAGNEZIX®) demonstrates equivalent clinical and radiographic outcomes to standard titanium screws in hallux valgus surgery.

## Material and methods

### Ethical approval

This prospective, randomized controlled study (according to EN ISO 14155–1:2009 and EN ISO 14155–2:2009) was approved by the ethics committee of medical school hannover, monitored by an independent trial center, and conformed to the principles in the Declaration of Helsinki. All participating patients provided voluntary written informed consent.

### Implants

**Figure 1:**
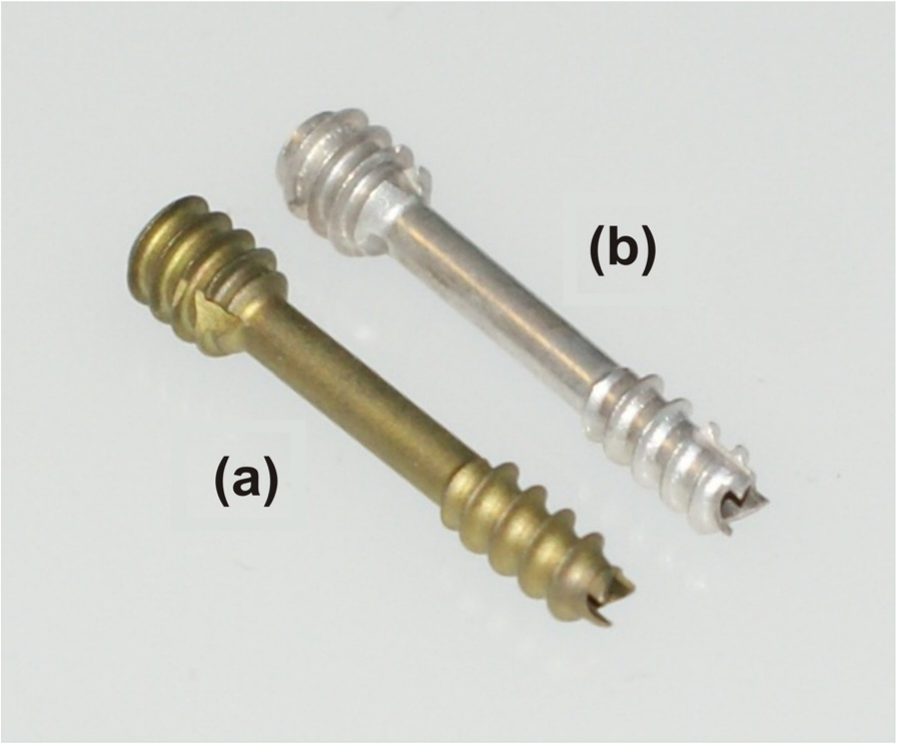
**The two cannulated screws with the same design. a)** The titanium screw (Fracture compressing screw, Königsee Implantate GmbH, Am Sand 4, 07426 Allendorf, Germany), **b)** MAGNEZIX® Compression Screw (Syntellix AG Schiffgraben 11, 30159 Hannover, Germany).

The ends of the cannulated screws (shaft Ø, 2.0 mm; cannulation Ø, 1.3 mm) included two threads (Ø 3.0 and 4.0 mm) with different pitches in order to achieve interfragment compression [13]. The implants were made of a powdered metallurgically processed magnesium alloy: this aluminum-free material consists of MgYREZr (a material similar to WE43) that contains >90 wt% magnesium. With an average grain size of <5 μm, this high-performance alloy demonstrates an offset elastic limit of R_p0.2_>250 MPa, tensile strength >275 MPa, and percent elongation at break >10%. Titanium screws with the same dimensions were used as the control (Figure 1).

### Study design

Between March 2010 and July 2011, 26 patients (26 feet) with symptomatic hallux valgus were enrolled in this study (Table 1). The inclusion and exclusion criteria are shown in Table 2. Patients were randomly assigned by the independent trial center to either group before implantation without the knowledge of the medical investigators or surgeons. The study protocol included eight study visits (V1, preoperation; V2, operation; V3, 1–3 days postoperation; V4, 4–8 days postoperation; V5, 2 weeks postoperation; V6, 6 weeks postoperation; V7, 3 months postoperation; V8, 6 months postoperation). Clinical examinations were performed at V1 and V3–8 and included determination of the range of motion (ROM) of the first metatarsophalangeal joint (MTPJ), American Orthopaedic Foot and Ankle Society (AOFAS) score for hallux, pain level according to the visual analog scale (VAS), satisfaction rate (very satisfied, satisfied, or unsatisfied with the results), and identification of any complications. Laboratory analyses (also performed at V1 and V3–8) included determination of magnesium levels in the blood and urine, standard electrolytes (e.g., potassic, sodium, chloride, calcium, and phosphate), renal parameters (e.g., urea, creatinine, and creatinine clearance) and liver parameters (e.g., GOT, GPT, GammaGT, and alkaline phosphatase).

**Table 1:** Demographic informations of the study groups

	**Degradable implantgroup (DI)**		**Titan implantgroup (TI)**	
	mean	SD	mean	SD
n	13		13	
gender [w/m]	11/2		13/0	
age [years]	57.2	7.2	49.9	16.5
weight [kg]	74.5	11.4	70.5	14.4
height [m]	1.68	7.0	1.68	9.1
BMI [kg/(m)^2^]	26.0	3.0	25.0	3.6

**Table 2:** Inclusion and exclusion criteria of the study

**Inclusion**	**Exclusion**
Symptomatic bunion with radiographic correlates	Operations on the symptomatic foot in the past
Patients aged 40-79	BMI> 32
Female fertile patients: obligate practice of two different secure contraceptive methods	Pregnancy or lactation
Normal function of the lower extremity	Neurological pathologies
	Bone mineral density abnormalities (e.g. radiographic detected bone cysts in the first ray, manifested osteoporosis)
	Allergies against study products (components of the screws)
	Substitution of magnesia or manifested hepato-renal diseases with possibel resulting bone mineral density abnormalities
	Participation in other studies 30 days before the start of this study and during the participation in this study

### Surgical technique

A high tourniquet was placed at the thigh. Lateral release was performed over an incision that was placed between the first and second metatarsal bones. The musculus adductor hallucis tendon was released, and the lateral sesamoid was mobilized. The medial approach was performed according to the technique described by Waizy et al. [14]. The exostoses were removed, and the center of the metatarsal head was marked with a 1.2 mm K-wire. An oscillating saw was used to perform a 90° chevron osteotomy. The distal fragment was displaced to the lateral side, and osteosynthesis was performed according to the patient group. Temporary fixation with a threaded K-wire (1.2 mm) was performed at the desired position of the screw. Intraoperative X-rays were acquired to verify correct K-wire positioning and determine screw length. A two-step pilot drillbit was used to make the countersunk hole in the head.

In the titanium group (TI), the compression screw was turned to generate compression, and the K-wire was removed after the screw was positioned. In the degradable group (DI), additional predrilling was performed using a 2.0 mm diameter hand-operated drill. The MgYREZr degradable compression screw was then inserted to generate compression, and the K-wire was removed after the screw was positioned.

The overriding bone of the proximal metatarsal fragment was removed using an oscillating saw. The tourniquet was released, and the skin was sutured. Postoperative bandages were applied by holding the big toe in the correct position.

### Radiography

All radiographs were conducted under standardized, weight-bearing conditions at V1, V3, and V6–8. Posterior-anterior radiographs were used to measure the hallux valgus angle (HVA), intermetatarsal angle (IMA), and the distal metatarsal articular angle (DMAA). To determine the axis of the first metatarsal bone, a line was drawn from the center of the head through the center of the base of the first metatarsal bone. This is considered the most precise, least biased method for determining postoperative effects [15]. All measurements were independently performed by three orthopedic surgeons.

### Postoperative treatment

The postoperative treatment regimens were the same for both groups. Postoperative dressings were removed between the first and third postoperative days (V1–3). The big toe was held and stabilized in the correct position using hallux valgus wool and crepe bandages for 6 weeks, and the bandages were changed twice a week. Physiotherapy with only passive MTPJ mobilization was initiated after the removal of the first dressings. Sutures were removed 2 weeks after surgery (V5). For 6 weeks, full weight-bearing mobilization was allowed when wearing an orthotic shoe with a stiff sole. High-impact sports were allowed after 12 weeks.

### Statistics

Significant differences in VAS and AOFAS scores for hallux were determined using *t* tests (IBM-SPSS version 20; Armonk, NY, USA). In this study, *p<*0*.*05 was considered statistically significant.

## Results

This clinical study was performed between March 2010 and February 2012 without interruption. Follow-up examinations were performed on 12 patients per group. One patient in each group dropped out of the study after surgery for personal reasons. Complications did not occur in any patients during the follow-up period.

**Figure 2:**
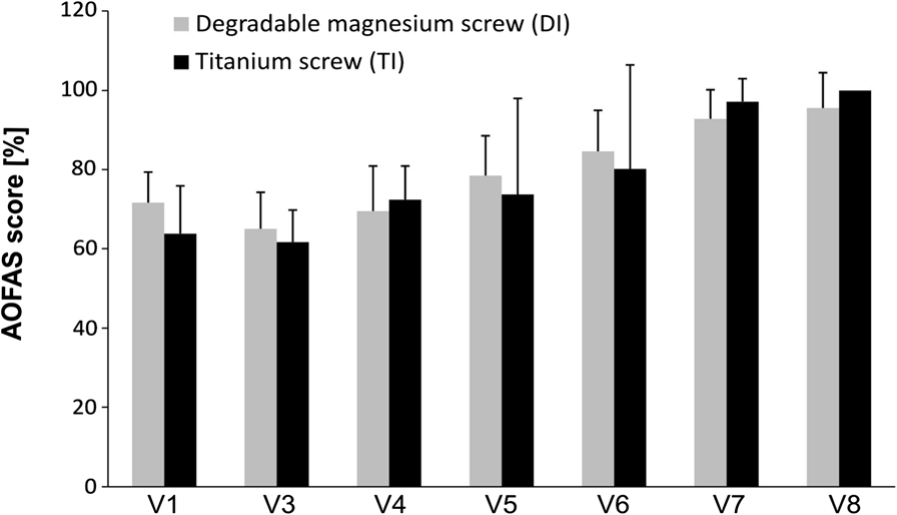
**Preoperativ (V1) and postoperative AOFAS score for hallux.** There is no significant difference between the improvement of the two groups (bars =mean value with standard deviation).

**Figure 3:**
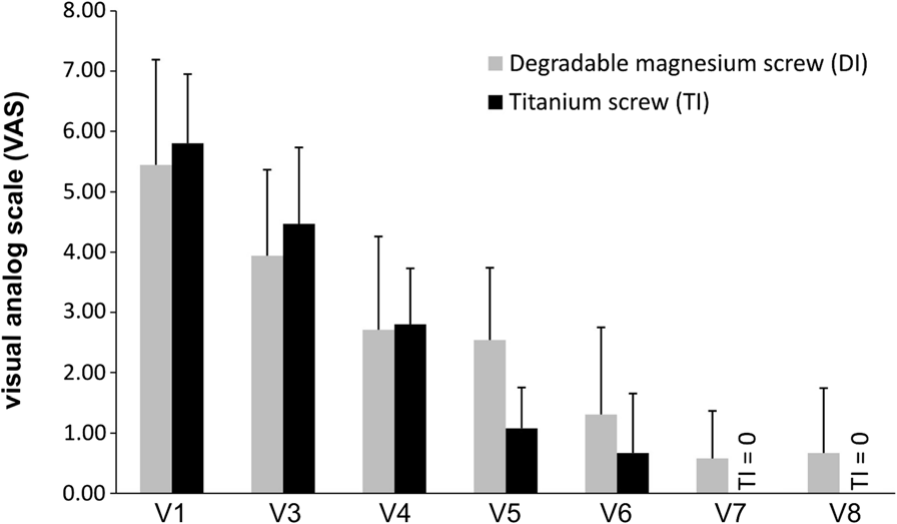
**Preoperativ (V1) and postoperative VAS scores.** There is no significant difference between the improvement of the two groups (bars = mean value with standard deviation).

Both groups demonstrated good to excellent results, including improvements in AOFAS score for hallux, and no significant differences were identified in any of the outcome measures (Figure 2). A total of 23 of 24 patients were very satisfied and indicated that they would undergo the same operation again. One patient in the DI group developed a superficial wound-healing problem and was unsatisfied. None of the patients developed a palpable gas cavity. VAS decreased in both groups (Figure 3). No significant differences were observed between visits.

MTPJ stiffness was not observed. All patients demonstrated minimum passive ROM of 60° and minimum active ROM of 50° at the MTPJ. The mean (standard deviation) operation time was 40.0 (9.1) minutes for the DI group and 34.0 (3.3) minutes for the TI group.

Chemical analysis revealed no significant elevations in blood magnesium levels. There were no significant differences between groups on the follow-up chemical or urine analyses.

**Figure 4:**
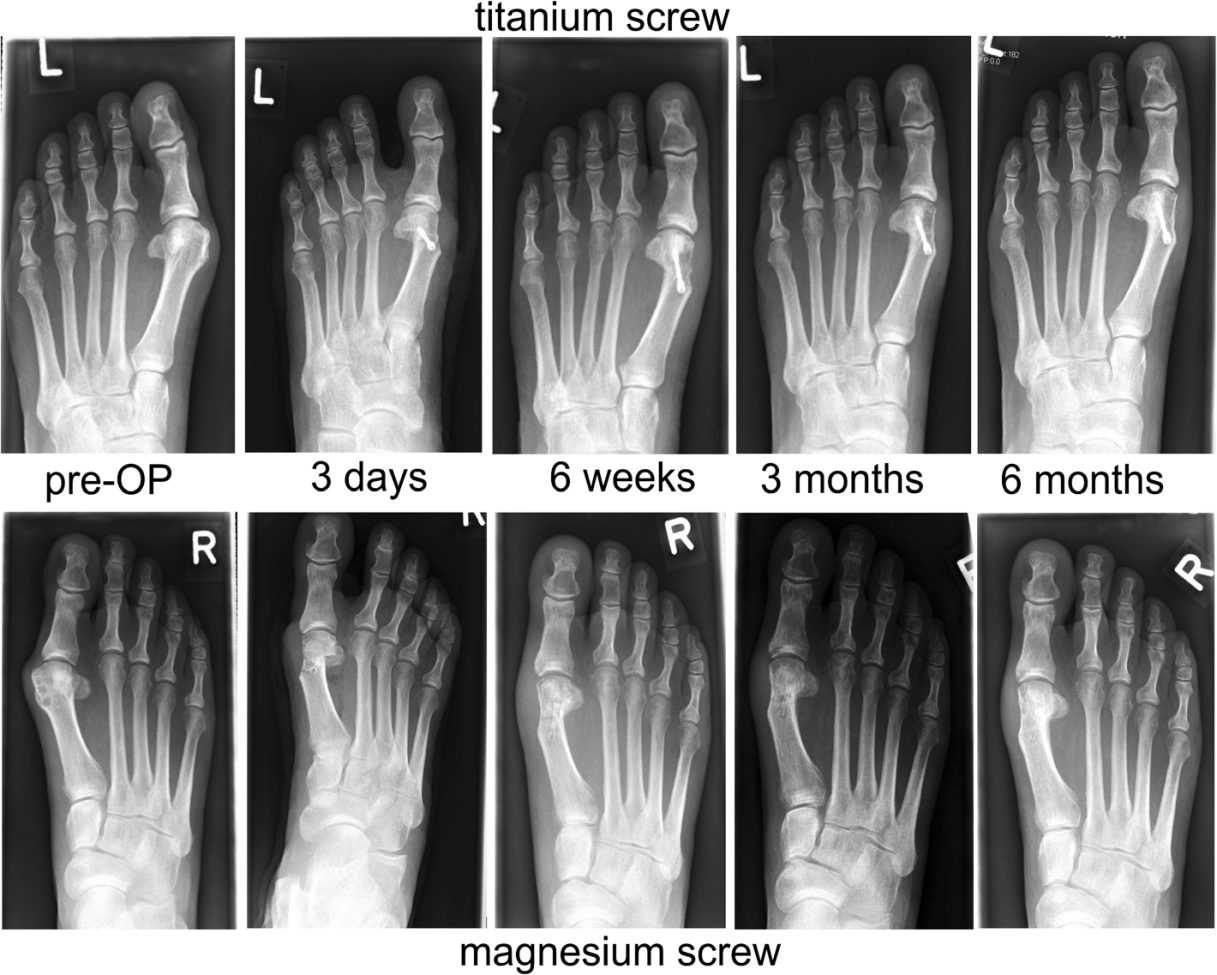
**Preoperative radiographs (posterior-anterior) of a mild hallux valgus deformity.** The correction is achieved by a chevron osteotomy. The postoperative radiographs show a bony healing in both groups.

HVA, IMA, and DMAA improved in both groups (Table 3), and the pre- and postoperative radiographs are shown in Figure 4. The postoperative X-rays demonstrated no signs of avascular necrosis, no bone erosion due to the development of gas cavities, and no advanced arthritis in the MTPJ. The healing rate was 100%. None of the screws had to be removed during the study. Although 1 patient in the TI group developed a symptomatic screw head, she refused to have the screw removed during the 6 month follow-up period for personal reasons. The screw was removed 8 months after implantation.

**Table 3:** The IMA, HVA and DMAA preoperative and postoperative after 6 months

		**Degradable implantgroup (DI)**		**Titan implantgroup (TI)**	
		mean	SD	mean	SD
IMA	Preop.	12.88	1.82	12.58	1.44
	6 months	7.67	2.89	6.04	2.49
HVA	Preop.	24.03	7.59	23.53	0.52
	6 months	16.19	8.93	11.76	6.41
DMAA	Preop.	11.01	5.05	12.91	6.96
	6 month	7.28	4.07	5.43	2.64

All adverse effects and complications were documented. General adverse events noted during this study included postoperative sickness (n=3, 2 DI and 1 TI patient) and pneumonia at 5 months after the operation (n=1 TI patient).

Three superficial wound complications developed and demonstrated delayed wound healing (2 DI patients and 1 TI patient). Infections were ruled out by laboratory analysis and clinical inspection. All 3 wounds healed without revision. No allergic reactions or further systemic reactions were observed in either group. Complex regional pain syndrome (CRPS) and severe adverse effects were not observed.

## Discussion

The chevron osteotomy has shown good to excellent clinical results in mild to moderate hallux valgus, with high patient satisfaction rates. Those results were mostly based on Level 3–4 studies [16]. In this prospective randomized study concerning the screw, both groups showed good to excellent clinical and radiographic results with a high satisfaction rate. The equivalent clinical outcomes were considered a result of the operative procedure, and we did not detect any influence of the implant on the clinical features.

The degradation of an implant can necessitate an undesirable second operative therapy for implant removal. Besides the additional cost, further problems may develop due to elevated infection risk associated with implant removal [17]. Also, up to 20% of patients develop new symptoms after a second surgery to remove the implant [18]. Coughlin reported that additional operative procedures for implant removal (screws, plates, pins) were necessary in 15% of patients with hallux valgus [19]. In the present study, no implant removals were necessary during the first 6 months after surgery. This may be attributable to the design of the screw, which lacked a prominent screw head. The implant removal rate due to soft tissue irritation was reported to be low with the head design of the Herbert screw [20]. However, because this study had a relatively short follow-up period and reduced irritation due to the screw head design, it was difficult to show the potential benefit of degradable screws given the reduction in redundant surgery.

Degradable implants are currently in clinical use for fixation in chevron osteotomy. The clinical outcome is excellent for both degradable and nondegradable implants. Caminear et al. retrospectively studied a series of 18 chevron osteotomies fixed with copolymer pins and observed high AOFAS scores (87.4±14.9) and only one giant cell granuloma [21]. The AOFAS score for hallux is not validated; however, it is the most used clinical scoring system. Small et al. conducted a retrospective study of 71 chevron osteotomies fixed with degradable polymer pins and reported a 100% bone healing rate, but osteolysis appeared in 5.6% of cases [22]. The only previous comparative study (chevron osteotomy: polymer pins vs. permanent K-wires) was reported by Gill et al. [23]. No differences were found regarding the prevalence of clinical symptomatic complications, but osteolysis was observed in 10.2% of patients in the polymer-based group. In our study, 3 (2 DI and 1 TI) patients experienced delayed wound healing. Further studies with a greater number of patients are necessary to identify a possible clinical difference.

Stable fixation with accelerated bone healing reduces immobilization time and the risk of developing joint stiffness. In contrast to polymer-based implants, magnesium alloys showed promising biomechanical results *in vitro*[24,25] and *in vivo*[26]. The in vivo results also attributed an osteoconductive quality to the magnesium alloy MgYREZr [8]. This would facilitate early bone healing, and consequently, faster mobilization with early recovery, which would potentially benefit the patients. Further studies should test this osteoconductive hypothesis by measuring bone healing velocity.

The use of degradable magnesium implants is controversial. The degradation process produces hydrogen gas, and gas cavities have been described. The source of the gas cavities remains an issue of debate [27]. In clinical use, gas formation would be an obstacle to bone and wound healing. Waizy et al. performed a rabbit study with a 1 year follow-up to test the alloy MgYREZr and found no bone erosion due to gas cavities [8].

Corrosion occurs in both degradable and permanent implants. Implant wear may cause the accumulation of particles around implants that stimulate inflammation, osteoclast activation, and osteoblast inhabitation. This pathway induces osteolysis and may also trigger hypersensitivity and allergic reactions [28]. Witte et al. previously demonstrated that the magnesium alloy WE43 was nonallergenic in an epicutaneous patch test [29]. MgYREZr is similar to WE43; therefore, we hypothesized that it may also have a nonallergic composition. We did not observe any cases of allergic reaction during follow-up.

The degradation of a magnesium alloy can potentially induce a systemic inflammatory reaction or pathologic changes in visceral organs. However, to date, no *in vivo* studies have reported either of those adverse events [30]. Waizy et al. postulated that MgYREZr would have good biocompatibility due to the absence of acute, subacute, and chronic systematic inflammatory reactions and the absence of specific pathologic changes in the visceral organs in an *in vivo* study [8]. The present clinical study confirmed the good clinical outcome hypothesized for this degradable magnesium alloy.

The primary limitation of this study was the relatively short follow-up time, and future studies should be focused on long-term consequences. We set the endpoint of the study at 6 months follow-up because this was the typical endpoint for operative hallux valgus therapy at our institution. At 6 months, complete bone healing should be attained, and further radiographic controls should not be necessary. We were not able to verify complete screw degradation; however, based on *in vivo* results from Waizy et al., it is reasonable to assume that the magnesium alloy was completely or nearly completely degraded [8]. Another limitation of our study was the relatively low statistical power of the radiographic measurements. However, we could show an equivalent clinical outcome, and we did not observe pseudarthrosis or other implant-associated complications. The strength of this study is that it is the first prospective, randomized, single surgeon study to investigate a degradable, magnesium-based implant.

## Conclusion

This pilot study demonstrated that the degradable magnesium-based screw was radiographically and clinically equivalent to the conventional titanium screw. We did not observe any instances of foreign body reaction, osteolysis, or systemic inflammatory reaction. Larger prospective randomized trials with a longer follow-up are needed to confirm the findings of this study.

## Abbreviations

AOFAS: American Orthopaedic Foot and Ankle Society; DI: Degradable group; DMAA: Distal metartarsal angle; HVA: Hallux valgus angle; IMA: Intermetatarsal angle; MTPJ: Metatarsophalangeal joint; ROM: Range of motion; TI: Titanium group; VAS: Visual analog scale.

## Competing interests

Mr. Robert Schavan is employed by the company Syntellix; he has not influenced the collection of data or its interpretation. The other authors have no competing interests.

## Authors’ contributions

HW and CSC initiated the study and participated in its design and coordination. KR and UK collected and analyzed the data. AW and JD helped to draft the manuscript. YN coordinated the clinical study. RS participated in the design of the study and contributed the screw samples. HaW initiated and performed the study, analyzed the data, and wrote the manuscript. All authors read and approved the final manuscript.
