# Correction to: Biodegradable magnesium-based screw clinically equivalent to titanium screw in hallux valgus surgery: short term results of the first prospective, randomized, controlled clinical pilot study

**DOI:** 10.1186/s12938-020-00818-8

**Published:** 2020-11-23

**Authors:** Henning Windhagen, Kerstin Radtke, Andreas Weizbauer, Julia Diekmann, Yvonne Noll, Ulrike Kreimeyer, Robert Schavan, Christina Stukenborg-Colsman, Hazibullah Waizy

**Affiliations:** 1 Department of Orthopaedic Surgery, Hannover Medical School, Anna-von-Borries-Str.1-7, 30625 Hannover, Germany; 2 Syntellix AG, Schiffgraben 11, 30159 Hannover, Germany

## Correction to: BioMedical Engineering OnLine (2013) 12:62 10.1186/1475-925X-12-62

It was highlighted that in the original article [1] the below two errors were detected:

In the study the Kitaoka Score was used for the clinical recording of the function and pain situation of the foot in the study. This enabled the assessment of the level of activity and the pain situation of the patients. The score was used consistently in all patients in the study, both preoperatively and postoperatively. In summary, all examinations were carried out uniformly and comparable with the Kitaoka score. However, in the publication, the score was named as AOFAS Score - we apologize for this error. This has no influence on the informative value of the study.

The graphic (Fig. 2) of the publication for visit points V5 and V6 is based on data collection within the study. The graphics were created by editing the entered data tables. Unfortunately, in this process there was a transmission error. The different data values only affected patient No. 34 and only the values at V5 and V6. This did not result in any difference in the significance tests and the conclusion of the study is not affected by this.

**Fig. 2:**
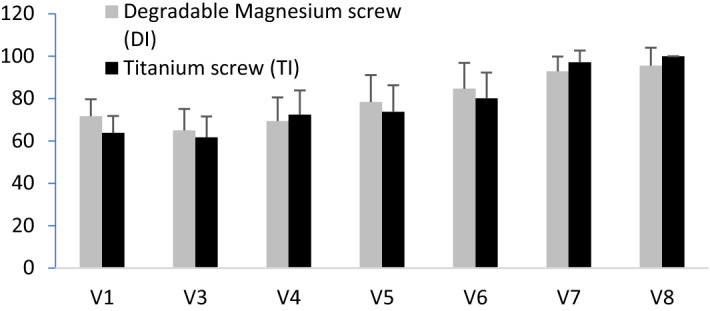
Preoperative (V1) and postoperative Kitaoka score for hallux. There is no significant difference between the improvement of the two groups (bars =mean value with standard deviation)
